# A new definition of left ventricular compaction/noncompaction - the new gold-standard?

**DOI:** 10.1186/1532-429X-15-S1-O13

**Published:** 2013-01-30

**Authors:** Gaby Captur, Andrew Flett, Andrea Barison, Robert Wilson, Daniel Sado, Christopher Cook, William J McKenna, Timothy J Mohun, Vivek Muthurangu, Perry Elliott, James Moon

**Affiliations:** 1 Division of Cardiovascular Imaging, The Heart Hospital part of University College London NHS Foundation Trust, London, UK; 2 Department of Developmental Biology, MRC National Institute for Medical Research, London, UK; 3 Scuola Superiore Sant' Anna, Fondazione "G. Monasterio" CNR, Regione Toscana, Italy; 4 UCL Centre for Cardiovascular Imaging, Great Ormond Street Hospital for Children, London, UK; 5 UCL Institute of Cardiovascular Science, University College London, London, UK

## Background

Abundant, abnormal myocardial trabeculae define left ventricular noncompaction (LVNC) but measurement is difficult and at least 5 techniques are described. We hypothesized that part of the reason for difficulties was that LV trabeculae were fractal in nature and beyond the simple geometry of 1 or 2 dimensional (2D) measurement. We designed and validated a new, rapid, clinically applicable method of measuring LV trabeculae based on fractal analysis.

## Methods

We developed a fractal analysis technique for measuring LV trabeculation using CMR volume stack images.

**Figure 1:**
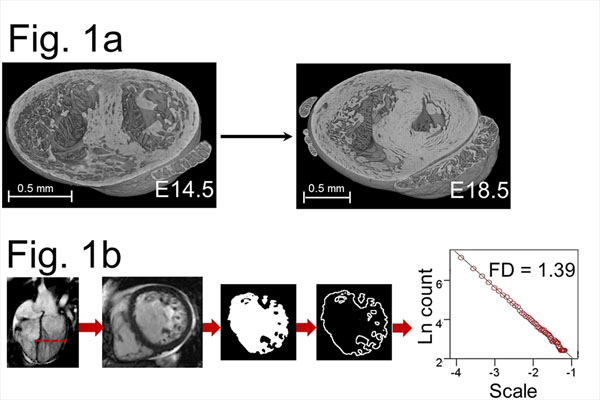
(a) Maturation of the murine heart as seen with HREM (E14.5 to E18.5). (b) Image processing sequence of human CMR data. Each slice from within the LV cine stack undergoes binarization, edge-detection and fractal analysis (illustrative LVNC heart shown). HREM indicates high-resolution episcopic microscopy; E, embryonic day; CMR, cardiovascular magnetic resonance; LV, left ventricle; LVNC, left ventricular noncompaction; Ln, logarithm.

With no gold-standard for LV trabeculae, we validated the method on the actual compaction process itself using 3D images of the compacting embryonic murine heart (between day 14.5 and 18.5) during cardiomorphogenesis (Figure-1a). We studied 24 mouse embryos, each with 1,000-1,200 ventricular slices in a 3D isotropic dataset (resolution 3 microns). In humans, hearts were analyzed for slice-by-slice fractal gradients (Figure-1b). Here we compared health to overt disease (LVNC) and compared the trabeculae of black and white healthy volunteers. Intra and inter-observer reproducibility of 60 fractal readings was analyzed and compared with 2 other CMR approaches (Petersen and Jacquier). In humans we studied a total of 135 subjects: LVNC cases, n=30; healthy blacks, n=30; healthy whites, n=75.

## Results

**Figure 2:**
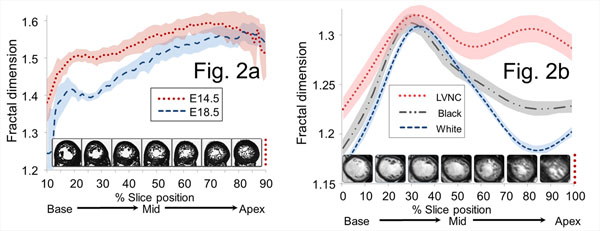
(a) In embryonic mice, transformation from trabeculated to more compacted myocardium is accompanied by a fall in FDs across the left ventricle from base to apex (shaded areas represent mean ± 95% CI). (b) In humans studied by CMR, FDs change in a characteristic pattern across the left ventricle in LVNC, healthy black and healthy white volunteers (shaded areas represent s.e.m.). FD indicates fractal dimension; CI, confidence intervals; s.e.m., standard error of mean. Other abbreviations as in Figure 1.

The fractal approach could measure embryonic compaction revealing a fall in FD with cardiac development as the heart compacts (E14.5 to E18.5, P<0.0001)(Figure-2a).

All 135 human hearts were analyzable (average analysis time: 5.3±0.4 minutes per subject). The FDs of whole human hearts were: LVNC, 1.29±0.007; healthy black, 1.25±0.006; healthy white, 1.23±0.003, P value <0.001 for trend and pairwise comparisons. Across the heart there was a characteristic base-to-apex FD gradient. This was lost in LVNC (Figure-2b) so the maximal difference was noted in the apical third (maximal apical FD: LVNC, 1.391±0.010; black volunteers, 1.253±0.005; white volunteers, 1.235±0.004; P<0.0001). A maximal apical FD cut-off of ≥1.30, predicted LVNC with a high degree of accuracy, AUC 1.0. Normal reference ranges were created for black and white populations. Reproducibility analysis showed the fractal technique to be substantially more reproducible than other CMR methods.

## Conclusions

A fractal-based approach to measuring LV trabeculae is mathematically sound, reproducible, clinically feasible and for the first time, validated against embryonic myocardial compaction. It describes trabeculation as a novel continuous variable, distinguishing health from disease but also detecting more subtle inter-ethnic differences.

## Funding

J.C.M is supported by the Higher Education Funding Council for England.

T.M. is supported by funding from the Medical Research Council (U117562103).

Funding for development of high-resolution episcopic microscopy of embryos (http://www.embryoimaging.org) was provided by the Wellcome Trust (WT087743MA).

G.C. is supported by the University College London through a Graduate Research Scholarship and by the European Union through a Science and Technology Grant.

This work was undertaken at the University College London Hospital and University College London, which receive a proportion of funding from the Department of Health's National Institute for Health Research Biomedical Research Centres funding scheme.

